# Integration of mental health services into HIV healthcare facilities among Thai adolescents and young adults living with HIV

**DOI:** 10.1002/jia2.25668

**Published:** 2021-02-10

**Authors:** Tavitiya Sudjaritruk, Linda Aurpibul, Wipaporn Natalie Songtaweesin, Assawin Narkpongphun, Paul Thisayakorn, Tawalchaya Chotecharoentanan, Rachaneekorn Nadsasarn, Prapaporn Janjing, Chutima Saisaengjan, Thanyawee Puthanakit, the Mental Health Study Group

**Affiliations:** ^1^ Department of Pediatrics Faculty of Medicine Chiang Mai University Chiang Mai Thailand; ^2^ Clinical and Molecular Epidemiology of Emerging and Re‐emerging Infectious Diseases Research Cluster Faculty of Medicine Chiang Mai University Chiang Mai Thailand; ^3^ Research Institute for Health Sciences Chiang Mai University Chiang Mai Thailand; ^4^ Center of Excellence for Pediatric Infectious Diseases and Vaccines Chulalongkorn University Bangkok Thailand; ^5^ Department of Psychiatry Faculty of Medicine Chiang Mai University Chiang Mai Thailand; ^6^ Department of Psychiatry Faculty of Medicine Chulalongkorn University Bangkok Thailand; ^7^ Department of Pediatrics Faculty of Medicine Chulalongkorn University Bangkok Thailand

**Keywords:** anxiety disorders, depressive disorders, integrated mental health services, psychiatric disorders, suicidality, youth living with HIV

## Abstract

**Introduction:**

To assess the burden of depression, anxiety and suicidality; and to determine the impact of integrated mental health and HIV services on treatment outcomes among Thai adolescents and young adults living with HIV (AYHIV).

**Methods:**

A multicentre prospective cohort study was conducted among AYHIV (15 to 25 years), and age‐ and sex‐matched HIV‐uninfected adolescents and young adults (HUAY). The Patient Health Questionnaire 9‐item (PHQ‐9) and Generalized Anxiety Disorder 7‐item scales (GAD‐7) were used as screening tools for depressive and anxiety symptoms respectively. History of lifetime and recent suicidal ideations/attempts were ascertained. Elevated mental health screening scores were defined as having either significant depressive symptoms (PHQ‐9 ≥9), significant anxiety symptoms (GAD‐7 ≥10) or suicidality (lifetime; and recent [within two weeks]). Participants meeting these criteria were referred to psychiatrists for confirmatory diagnosis and mental health services. Follow‐up assessment with PHQ‐9 and GAD‐7 was performed one year after psychiatric referral.

**Results:**

From February to April 2018, 150 AYHIV and 150 HUAY were enrolled, median age was 19.0 (IQR:16.8 to 21.8) years and 56% lived in urban areas. Among AYHIV, 73% had HIV RNA <50 copies/mL, and median CD4 count was 580 (IQR:376 to 744) cells/mm^3^. At enrolment, 31 AYHIV (21%; 95%CI:14% to 28%) had elevated mental health screening scores; 17 (11%) significant depressive symptoms, 11 (7%) significant anxiety symptoms and 21 (14%) suicidality. Seven AYHIV (5%) had all three co‐existing conditions. These prevalences were not substantially different from HUAY. Urban living increased risk, whereas older age decreased risk of elevated mental health screening scores (*p* < 0.05). All AYHIV with elevated mental health screening scores were referred to study psychiatrists, and 19 (13%; 95%CI: 8% to 19%) had psychiatrist‐confirmed mental health disorders (MHDs), including adjustment disorder (n = 5), major depression (n = 4), anxiety disorders (n = 2), post‐traumatic stress disorder (n = 1) and mixed MHDs (n = 4). One year after psychiatric referral, 42% of AYHIV who received mental health services demonstrated an absence of significant mental health symptoms from the reassessments, and 26% had an improved score.

**Conclusions:**

With the significant burden of MHDs among AYHIV, an integration of mental health services, including mental health screenings, and psychiatric consultation and referral, is critically needed and should be scaled up in HIV healthcare facilities.

## 1 INTRODUCTION

Mental health disorders (MHDs) are one of the most prevalent non‐AIDS‐related comorbidities among people living with HIV (PLHIV). Several studies indicate elevated rates of MHDs in this population, including youth with perinatally or horizontally acquired HIV infection and adults living with HIV [1, 2, 3, 4, 5, 6, 7]. The global magnitude of depressive disorder is reported at 13% to 38% [8, 9, 10, 11, 12], anxiety disorders 15% to 32% [3, 13, 14] and suicidal behaviours 9% to 32% among adults living with HIV [15, 16, 17, 18, 19]. MHDs and HIV infection are closely interlinked. Underdiagnosed and untreated MHDs are significant impediments to successful HIV treatment outcomes [20, 21, 22]. Thus, the integration of mental health services into HIV healthcare facilities is critically needed.

Adolescents and young adults living with HIV (AYHIV) are a vulnerable population for MHDs because of the impact of HIV on physical and pubertal growth, psychological stress and HIV‐related comorbidities [23, 24, 25, 26]. Existing evidence from resource‐rich and resource‐limited countries indicate high prevalence of MHDs in AYHIV [27, 28, 29, 30, 31]. The Adolescent Medicine Trials Network for HIV/AIDS Interventions reported a 21% prevalence of depression, and 14% prevalence of anxiety among AYHIV [27]. Similarly, a Kenyan study demonstrated that the prevalence of major depressive disorder (MDD), anxiety disorders and suicidal risk was 18%, 32% and 18% respectively [29]. Older age, alcohol use, stressful life events (e.g. change of primary caregiver, family member death, school failure) and HIV‐related stigma have previously been reported as contributing factors for MHDs in this population [32]. However, there are few data highlighting the burden and the associated factors of MHDs among Asian AYHIV due to limited availability of adolescent‐friendly mental health services, and insufficiency of infrastructure and resources to detect mental illnesses [33].

Currently, several international guidelines recommend universal mental health screening and treatment services for people with chronic diseases [34, 35, 36]. One well‐established clinical practice guideline is recommended by the Cystic Fibrosis Foundation, which suggests the annual screening of depression and anxiety disorders in adolescents and adults with cystic fibrosis, using the Patient Health Questionnaire‐9 (PHQ‐9) and the Generalized Anxiety Disorder 7‐item (GAD‐7) scale [36]. This guideline also recommends a stepped care model to provide initial clinical management, psychiatric referral and psychological and/or pharmacological interventions for patients with an abnormal mental screening [36]. There are as of yet no well‐established guidelines for delivering mental health services in HIV healthcare facilities.

Given the current lack of clinical guidance on integration of mental health and HIV services for AYHIV in Asian countries, this study set out to assess the burden of depression, anxiety disorders and suicidality, and to determine the impact of integrated services on mental health and HIV treatment outcomes among Thai AYHIV.

## 2 METHODS AND MEASUREMENTS

### 2.1 Study design and participants

We conducted a multicentre prospective cohort study among two paediatric HIV centres in Thailand, the Faculty of Medicine, Chiang Mai University (CMU) and the Faculty of Medicine, Chulalongkorn University (CU). AYHIV (both perinatally and horizontally acquired infections) who aged 15 to 25 years, were fully disclosed of HIV status, and received treatment services at both centres were enrolled. HIV‐uninfected adolescents and young adults (HUAY), including siblings, peers and neighbours of AYHIV and patients attending general paediatric clinics who had similar age and sex with AYHIV (ratio 1:1) were recruited from the same centres. Participants receiving treatments and/or interventions for psychiatric disorders, or had cognitive impairment or a condition which could compromise their ability to respond to study questionnaires and tools were excluded. This study was approved by the Research Ethics Committee, Faculty of Medicine, CMU, and the Institutional Review Board, Faculty of Medicine, CU. All participants provided written informed consent prior to study enrolment; caregiver consent was obtained for participants aged <18 years.

### 2.2 Clinical assessments and data collection

Sociodemographic and HIV‐related characteristics of eligible participants were extracted from electronic medical records. Sex (male vs. female) and sexual orientation (heterosexual vs. lesbian, gay, bisexual, transgender or queer [LGBTQ]) were self‐evaluated. We also assessed risk‐taking behaviours with the HEEADSSS (home environment, education, employment, activities, drug abuse [alcohol and illicit drugs], sexuality, suicidality and safety from injury and violence) review of systems tool [37]. Stressful life events, including breaking up with boyfriend/girlfriend/sex partner, family member death, school failure/suspension, unemployment and other unpleasant life experiences within 12 months were collected using a study questionnaire. Alcohol consumption and alcohol‐related problems were assessed by the Alcohol Use Disorders Identification Test (AUDIT), a 10‐item screening tool with a score range from 0 to 40. AUDIT scores ≥8 indicate hazardous/harmful alcohol use [38].

### 2.3 Mental health screening and assessments

In this study, the PHQ‐9, a brief, validated, self‐administered questionnaire based on the Diagnostic and Statistical Manual of Mental Disorders, 4th edition (DSM‐IV) criteria for MDD, was used to screen for depression. For each question, scoring was 0 (not at all) to 3 (nearly every day), with a total score range of 0 to 27 [39]. Participants with PHQ‐9 scores ≥9 were defined as having significant depressive symptoms [39, 40]. The GAD‐7, a brief, validated, self‐reported screening tool based on DSM‐IV criteria for GAD, was used to identify anxiety disorders. Each of the seven questions was scored from 0 (not at all) to 3 (nearly every day), with total scores ranging from 0 to 21 [41]. Participants with GAD‐7 scores ≥10 were defined as having significant anxiety symptoms [41, 42]. Suicidality was assessed by question #9 of PHQ‐9 asking about a recent suicidal ideation within the past two weeks, and interview questions asking about lifetime suicidal ideation and/or attempts. Participants who had recent suicidal thoughts within two weeks, or had ever contemplated and/or attempted suicide were considered as having suicidality for the purposes of this study.

### 2.4 Definitions of elevated mental health screening scores

Elevated mental health screening scores were defined as having either significant depressive symptoms, significant anxiety symptoms or suicidality (lifetime and recent). Participants demonstrating elevated scores at enrolment were referred to a study psychiatrist for psychiatric assessments, including psychiatric interview, identification of psychiatric emergencies (e.g. suicidality), review of past psychiatric history, mental status examination and behavioural observation; and for a confirmatory diagnosis based on the DSM‐V criteria. Those with confirmed diagnoses were offered mental health services at the psychiatric clinic, including mental health counselling, psychotropic medications and psychological interventions, under the Thai national health insurance programme. Mental health outcomes were evaluated by the same psychiatrist during study follow‐up. At one year, PHQ‐9, GAD‐7 and recent suicidality (PHQ‐9, question #9), as well as CD4 T‐cell count and HIV RNA level were re‐assessed to determine mental health status and HIV treatment outcomes of all participants who had elevated mental health screening scores at enrolment and were linked to psychiatric services.

### 2.5 Statistical analysis

The prevalence of elevated mental health screening scores was calculated among AYHIV and HUAY. Univariable logistic regression analyses were performed to identify factors associated with elevated scores. Covariates demonstrating a *p* < 0.10 were included in the multivariable model. Magnitude of associations was summarized with crude (crude ORs) and adjusted odds ratios (aORs) for univariable and multivariable analyses respectively. The comparison of HIV treatment outcomes among AYHIV between enrolment and one‐year follow‐up was conducted using Wilcoxon‐signed rank test. All statistical analyses were carried out using Stata statistical software, version 14. (StataCorp LP, College Station, TX). A two‐sided *p* < 0.05 was taken to be statistically significant.

## 3 RESULTS

### 3.1 Sociodemographic characteristics and risk‐taking behaviours of study participants

From February to April 2018, 150 AYHIV and 150 age‐ and sex‐matched HUAY were enrolled. Median age was 19.0 (interquartile range [IQR]: 16.8 to 21.8) years. Twenty‐four participants (8%) were LGBTQ, and 276 (92%) were heterosexual. Approximately half (54%) had biological parents (mother, father or both) as primary caregivers, 56% lived in urban areas and 56% had household incomes ≥500 USD/month. At enrolment, 234 participants (78%) attended school, and 98 (33%) were employed in full‐ or part‐time jobs. One‐hundred and sixty‐four (55%) were current alcohol users; of whom 64 (39%) had AUDIT scores ≥8, and 16 (5%) had self‐reported illegal drug use. A comparison of sociodemographic characteristics and risk‐taking behaviours between AYHIV and HUAY is summarized in Table 1.

**Table 1:** Sociodemographic characteristics and risk‐taking behaviours of study participants

Characteristics^a^	Adolescents and young adults living with HIV (n = 150)	HIV‐uninfected adolescents and young adults (n = 150)	*p* ^b^
Age, years	18.7 (17.1 to 21.0)	19.4 (16.4 to 22.3)	0.43
Age <18 years	64 (42.7)	64 (42.7)	0.99
Sex			0.99
Male	75 (50.0)	75 (50.0)	
Female	75 (50.0)	75 (50.0)	
Sexual orientation			0.01
Heterosexual	132 (88.0)	144 (96.0)	
LGBTQ	18 (12.0)	6 (4.0)	
Primary caregiver			<0.001
Biological parents	57 (38.0)	104 (69.3)	
Grandparents	36 (24.0)	4 (2.7)	
Others	57 (38.0)	42 (28.0)	
Household income ≥500 USD/month	64 (42.7)	104 (69.3)	<0.001
Residential status			0.08
With family	132 (88.0)	138 (92.0)	
Group home/orphanage	13 (8.7)	12 (8.0)	
Others	5 (3.3)	0 (0)	
Urban living	78 (52.0)	91 (60.7)	0.13
School attendance	107 (71.3)	127 (84.7)	0.005
Full‐ or part‐time employment	62 (41.3)	36 (24.0)	0.001
Leisure activities			0.003
Playing sports	13 (8.7)	27 (18.0)	
Watching television/listening to music	47 (31.3)	53 (35.3)	
Playing computer/video games	10 (6.7)	16 (10.7)	
Playing cellphone	36 (24.0)	34 (22.7)	
Others	44 (29.3)	20 (13.3)	
Current smoker	20 (13.3)	24 (16.0)	0.51
Current alcohol use	73 (48.7)	91 (60.7)	0.04
Hazardous or harmful alcohol use (AUDIT scores ≥8)	28 (18.7)	36 (24.0)	0.26
History of illicit drug use	6 (4.0)	10 (6.7)	0.30
Having someone in sexual relationships	66 (44.0)	67 (44.7)	0.91
Ever contemplated or attempted suicide during lifetime	17 (11.4)	15 (10.0)	0.69

AUDIT, Alcohol Use Disorders Identification Test; LGBTQ, lesbian, gay, bisexual, transgender or queer; USD, US dollar.

^a^ Data were presented as n (%) for categorical data, and median (interquartile range) for continuous data

^b^ Data compared using Pearson’s chi‐squared test for categorical data, and Wilcoxon rank sum test for continuous data.

### 3.2 HIV‐related characteristics of AYHIV

Of 150 AYHIV, 141 (94%) had perinatally acquired HIV infection. At enrolment, all were receiving combination antiretroviral therapy (cART); 88 (59%) were nucleoside‐reverse transcriptase inhibitor‐based, 47 (31%) protease inhibitor‐based and 15 (10%) integrase strand transfer inhibitor‐based regimens. Median CD4 T‐cell counts were 580 (IQR: 376 to 744) cells/mm^3^, and 73% were virologically suppressed (HIV RNA <50 copies/mL). Twenty‐three percent reported missed doses of cART in the past three months. Median age at disclosure of HIV status was 12.0 (IQR: 10.1 to 13.7) years. Most of AYHIV (88%) had disclosed their HIV status to other family members, but only 22% to sex partners, and 13% to other people.

### 3.3 Stressful life events within preceding 12 months

Within the preceding 12 months of enrolment, 51 participants (17%) had experienced a relationship breakup, 44 (15%) had lost a family member and 16 (5%) had experienced a change in primary caregiver. Additionally, 15 (5%) had failed a school term/class, 8 (3%) were suspended from school and 13 (4%) were unemployed. There were also participants reporting experiencing physical abuse (n = 21; 7%), forced sex (n = 4; 1%), severe accidents (n = 11; 4%), witnessing physical violence (n = 19; 6%) and being arrested (n = 6; 2%). AYHIV had greater proportion of unemployed, whereas lower proportion of family deaths and failed school term/class, compared with HUAY (*p* < 0.05) (Table 2).

**Table 2:** Stressful life events within the preceding 12 months of study participants

Characteristics^a^	Adolescents and young adults living with HIV (n = 150)	HIV‐uninfected adolescents and young adults (n = 150)	*p* ^b^
Broke up with boyfriend/girlfriend/sex partner			0.38
Yes	26 (17.3)	25 (16.7)	
No	77 (51.4)	88 (58.7)	
Not applicable	47 (31.3)	37 (24.6)	
Family member death			<0.001
Yes	18 (12.0)	26 (17.3)	
No	68 (45.3)	110 (73.4)	
Not applicable	64 (42.7)	14 (9.3)	
Change of primary caregiver			0.06
Yes	11 (7.3)	5 (3.3)	
No	135 (90.0)	134 (89.4)	
Not applicable	4 (2.7)	11 (7.3)	
Failed a school term/class			0.001
Yes	5 (3.3)	10 (6.7)	
No	105 (70.0)	124 (82.7)	
Not applicable	40 (26.7)	16 (10.7)	
Suspended from school			0.002
Yes	4 (2.7)	4 (2.7)	
No	106 (70.7)	130 (86.7)	
Not applicable	40 (26.6)	16 (10.7)	
Unemployed			0.001
Yes	10 (6.6)	3 (2.0)	
No	64 (42.7)	41 (27.3)	
Not applicable	76 (50.7)	106 (70.7)	
Experienced physical abuse			0.82
Yes	10 (6.7)	11 (7.3)	
No	140 (93.3)	139 (92.7)	
Experienced forced sex			0.31
Yes	3 (2.0)	1 (0.7)	
No	147 (98.0)	149 (99.3)	
Experienced a severe accident			0.36
Yes	7 (4.7)	4 (2.7)	
No	143 (95.3)	146 (97.3)	
Witnessed physical violence			0.59
Yes	10 (6.7)	9 (6.0)	
No	139 (92.7)	141 (94.0)	
Not applicable	1 (0.6)	0 (0)	
Arrested			0.41
Yes	4 (2.7)	2 (1.3)	
No	146 (97.3)	148 (98.7)	

^a^ Data were presented as n (%)

^b^ Data were compared using Pearson’s chi‐squared test.

### 3.4 Prevalence of elevated mental health screening scores

Overall prevalence of elevated mental health screening scores was 20% (n = 59; 95% confidence interval [95%CI]: 15% to 24%), with no significant differences seen between AYHIV (n = 31; 21%; 95%CI: 14% to 27%) and HUAY (n = 28; 19%; 95%CI: 12% to 25%) (*p* = 0.66). Eleven participants (4%), seven of whom were AYHIV, had all three co‐existing conditions of significant depressive symptoms, significant anxiety symptoms and suicidality. LGBTQ participants tended to have higher prevalence of elevated mental health screening scores compared with heterosexual participants, though this comparison was not significant (33% vs. 18%; *p* = 0.08).

Of 300 adolescents, 34 had PHQ‐9 scores ≥9, corresponding to an overall prevalence of significant depressive symptoms of 11% (95%CI: 8% to 15%). The prevalence was the same between AYHIV and HUAY (11%; 95%CI: 6% to 17%). Nineteen participants had GAD‐7 scores ≥10, corresponding to an overall prevalence of significant anxiety symptoms of 6% (95%CI: 4% to 9%), which was not significantly different between AYHIV (7%; 95%CI: 3% to 12%) and HUAY (5%; 95%CI: 2% to 9%) (*p* = 0.48). Additionally, 40 participants demonstrated suicidality; 32 ever had lifetime suicidal ideations, 8 ever had lifetime suicidal attempts and 26 had a recent suicidal thoughts within two weeks, corresponding to an overall prevalence of suicidality of 13% (95%CI: 9% to 17%), which was also not different between groups (AYHIV:14% vs. HUAY:13%; *p* = 0.73). Notably, 68% of participants with elevated mental health screening scores reported suicidality.

### 3.5 Associated factors of elevated mental health screening scores

In multivariable analysis for AYHIV, residing in urban areas (aOR: 2.96; 95%CI: 1.11 to 7.86) increased the risk of elevated mental health screening scores, whereas older age (aOR: 0.78; 95%CI: 0.62 to 0.99 per one year increase in age) decreased the risk (Table 3). Elevated mental health screening scores tended to be associated with poorer cART adherence, but the association did not reach statistical significance (crude OR: 1.88; 95%CI: 0.78 to 4.53). Other HIV‐related characteristics and stressful life events did not demonstrate any significant associations (*p* > 0.05). For HUAY, no factors associated with elevated mental health screening scores were identified (*p* > 0.05).

**Table 3:** Associated factors of elevated mental health screening scores among adolescents and young adults living with HIV (n = 150)

Characteristics	Univariable analysis^a^			Multivariable analysis^a^		
	Odds ratio	95% confidence interval	*p*	Adjusted odds ratio	95% confidence interval	*p*
Sociodemographic characteristics						
Age (per 1 year increase)	0.85	0.72 to 1.01	0.07	0.78	0.62 to 0.99	0.04
Urban living (vs. rural living)	2.28	0.99 to 5.26	0.05	2.96	1.11 to 7.86	0.03
Hazardous or harmful alcohol use (AUDIT scores ≥8)	2.67	1.08 to 6.60	0.03	2.31	0.78 to 6.81	0.13
Stressful life events within 12 months						
Break up with boyfriend/girlfriend/sex partner	3.39	1.24 to 9.22	0.02	2.46	0.78 to 7.77	0.13
School suspension	13.74	1.35 to 139.36	0.03	11.57	0.97 to 137.75	0.05
Experienced physical abuse	4.38	1.18 to 16.26	0.03	2.39	0.49 to 11.59	0.28

AUDIT, Alcohol Use Disorders Identification Test.

^a^ Logistic regression analysis were performed. Covariates demonstrating a significance level of <0.10 in the univariable analysis were included in the multivariable model.

### 3.6 Psychiatric evaluations of participants with elevated mental health screening scores

**Figure 1:**
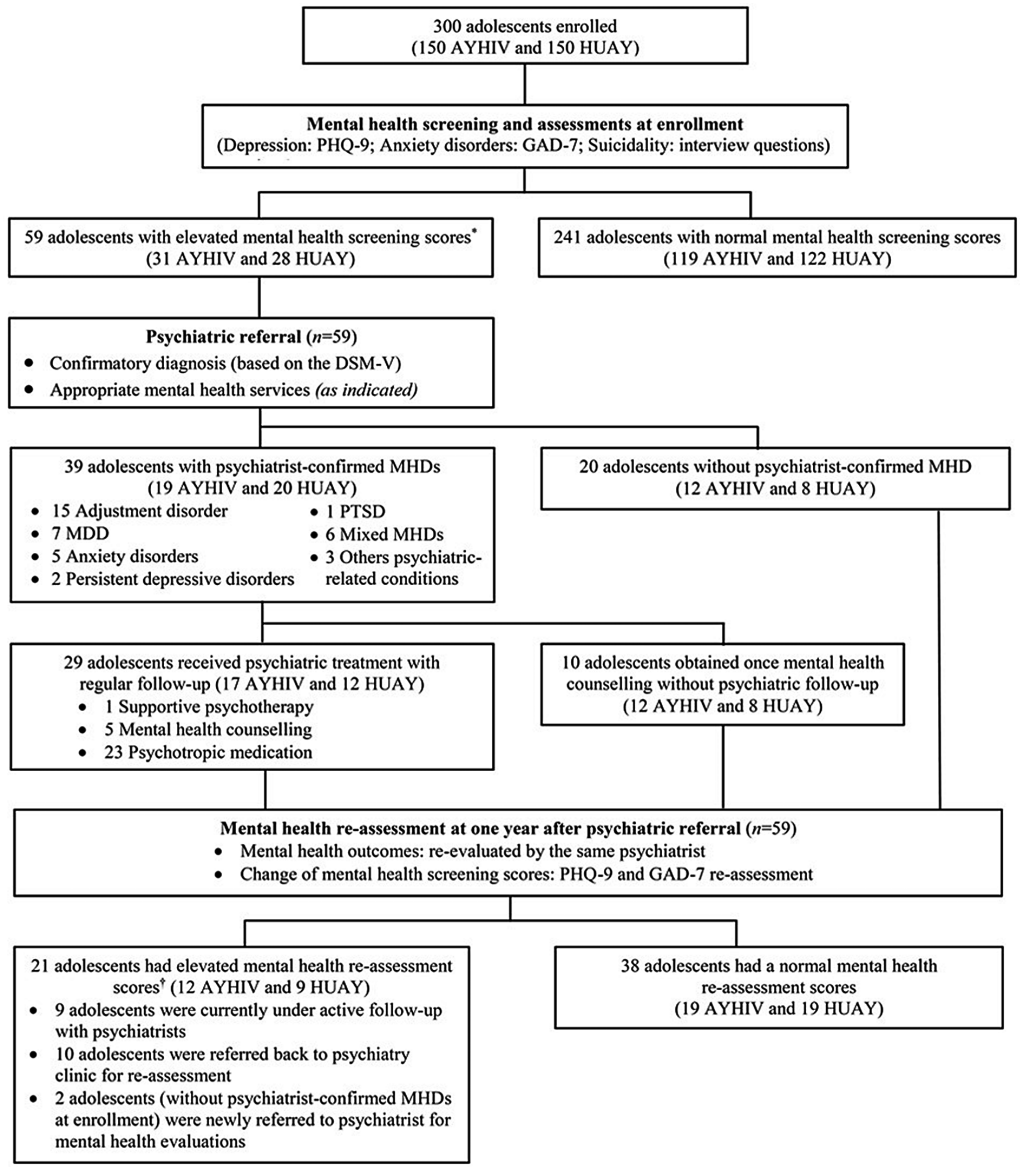
**Flow chart of study participants through the study**. Abbreviations: AYHIV, adolescents and young adults living with HIV; DSM‐V, the Diagnostic and Statistical Manual of Mental Disorders criteria, 5th edition; GAD‐7, the Generalized Anxiety Disorder 7‐item scales; HUAY, HIV‐uninfected adolescents and young adults; MDD, major depressive disorder; MHDs, mental health disorders; PHQ‐9, the Patient Health Questionnaire 9‐item; PTSD, post‐traumatic stress disorders. ^*^Elevated mental health screening scores was defined as having either significant depressive symptoms (PHQ‐9 ≥9), significant anxiety symptoms (GAD‐7 ≥10), or suicidal behaviours (ever had suicidal ideation and/or attempted suicide during lifetime, or had recent suicidal thought within the past 2 weeks [responded positively to PHQ‐9, question #9]). ^†^Elevated mental health reassessment scores was defined as having either significant depressive symptoms (PHQ‐9 ≥9, significant anxiety symptoms (GAD‐7 ≥10), or recent suicidal thought within the past 2 weeks (responded positively to PHQ‐9, question #9).

In this study, all 59 participants with elevated mental health screening scores (31 AYHIV and 28 HUAY) were referred to study psychiatrists (AN and PT) for confirmatory diagnosis and appropriate mental health services. Based on the DSM‐V criteria, 39 of 300 participants (13%; 95%CI: 9% to 17%), including 19 AYHIV (13%; 95%CI: 8% to 19%) and 20 HUAY (13%; 95%CI: 8% to 20%), had at least one MHD confirmed by a psychiatrist (Figure 1). MHDs included adjustment disorder (n = 15), MDD (n = 7), anxiety disorders (n = 5), persistent depressive disorder (PDD; n = 2), post‐traumatic stress disorder (PTSD; n = 1), mixed MHDs (n = 6) and other psychiatric‐related conditions (n = 3) (Figure 1) (Table S1).

All 39 participants with psychiatrist‐confirmed MHDs received appropriate mental health services at psychiatric clinic. Twenty‐nine (74%) received psychiatric treatment, including mental health counselling by a psychiatrist (n = 5), psychotropic medications (n = 23) and supportive psychotherapy (n = 1), with regular follow‐up, whereas 10 (26%) obtained once mental health counselling without psychiatric follow‐up (Figure 1). Among those receiving regular psychiatric follow‐up, 12 (41%) completed treatment course and were discharged from psychiatric clinic before their one‐year follow‐up visit, and 2 (7%) had incomplete follow‐up (Table S1; Figure S1).

The most frequently provided mental health service for participants with adjustment disorder was mental health counselling by psychiatrist (87%), and the most common psychotropic medications prescribed to participants with MDD, anxiety disorders, PDD, PTSD and mixed MHDs were selective serotonin reuptake inhibitors (SSRIs) and benzodiazepines (Table S1). During study follow‐up, one AYHIV with MDD and PTSD attempted suicide (Participant #31; Table S1).

### 3.7 Treatment outcomes at one‐year follow‐up after psychiatric referral

At one‐year follow‐up, all 59 participants with elevated mental health screening scores at enrolment were re‐evaluated with PHQ‐9, GAD‐7, and recent suicidality (PHQ‐9, question #9). Thirty‐eight participants (64%; 19 AYHIV and 19 HUAY) demonstrated an absence of significant mental health symptoms from the re‐evaluations, whereas 21 (36%; 12 AYHIV and 9 HUAY) had elevated scores. There were 2 of 20 participants (10%) without psychiatrist‐confirmed MHDs at enrolment demonstrating elevated scores at one‐year of follow‐up (Participants #41 and #56; Table S1). Of these 21 participants, nine were currently under active follow‐up with psychiatrists, 10 were referred back to psychiatric clinic for re‐assessment; and two were newly referred to psychiatrist for mental health evaluations (Figure 1) (Figure S1).

Among participants with psychiatrist‐confirmed MHDs (n = 39), 20 (51%), including 8/19 AYHIV (42%) and 12/20 HUAY (60%), exhibited an absence of significant mental health symptoms from the reassessments, whereas 5/19 (26%) AYHIV and 4/20 (20%) HUAY showed an improved score at one‐year follow‐up (Table S1). Among 19 AYHIV who had MHDs and received appropriate mental health services, their median CD4 T‐cell count (547 vs. 665 cells/mm^3^) and HIV RNA levels (40 vs. 40 copies/mL) were not different between enrolment and one‐year follow‐up (*p* > 0.05).

## 4 DISCUSSION

Approximately 20% of Thai AYHIV in this study demonstrated elevated mental health screening scores, of which 13% had psychiatrist‐confirmed MHDs. Younger age and urban living were associated with elevated scores. One year after appropriate mental health services, 42% of AYHIV with psychiatrist‐confirmed MHDs demonstrated an absence of significant mental health symptoms from the reassessments, and 26% showed an improved score. With the significant burden of MHDs among AYHIV, integration of mental health services into HIV healthcare settings should be implemented. Key resources include validated and culturally appropriate screening tools, well‐trained mental health counsellors, basic psychotropic medications and well‐established consultation/referral pathways between HIV and psychiatric clinics.

The prevalence of significant depressive symptoms among our Thai AYHIV was lower than those reported in African and US studies [27, 28, 29, 31]. A Malawian study found the prevalence of depressive symptoms of 19% among adolescents living with HIV aged 12 to 18 years, using the Children’s Depression Rating Scale, Revised (CDRS‐R) [31]. Similarly, a study in South African adolescents aged 13 to 19 years showed a 14% prevalence of depressive symptoms, evaluated by the Children’s Depression Inventory Short Form (CDI:S) [28]. Additionally, the US study demonstrated a 21% prevalence of depressive symptoms among AYHIV aged 12 to 24 years, using the Brief Symptom Inventory (BSI) [27].

Likewise, the prevalence of significant anxiety symptoms among our AYHIV was much lower than the prevalence of 25% observed in South African adolescents, using the Revised Children’s Manifest Anxiety Scale (RCMAS) [28], and the 32% prevalence seen in Kenyan youth, assessed by the Mini‐International Neuropsychiatric Interview for Children and Adolescents (MINI Kid) [29]. The prevalence of suicidality in our study was remarkably lower than the prevalence of 20% among Ugandan adolescents, measured by a questionnaire based on the ICD‐10 research diagnostic criteria [30], and the 24% prevalence among South African adolescents, measured by the MINI International Psychiatric Interview for Children and Adolescents Suicide Scale [28]. The between‐country variations in prevalence of depression, anxiety and suicidality observed may have been due to differences in age, sociodemographic characteristics, personal risk‐taking behaviours and HIV‐associated conditions of study samples; sociocultural factors of study settings; variations in mental health screening tools; and dissimilarities of HIV healthcare systems as well as mental health services among countries.

Unlike previous studies which commonly found the greater burden of MHDs among AYHIV than the general youth population [1, 43, 44, 45], our study demonstrated comparable prevalence of significant depressive and anxiety symptoms, as well as suicidality. We postulate this observation may have been because the majority of our AYHIV had perinatally acquired HIV infection, so have received HIV treatment services for long periods of time since early childhood. Therefore, these individuals are more likely to have had access to greater support, both structural and emotional, from their primary medical team. In contrast, general Thai youth tend to have fewer opportunities to access mental health assessment and management as adolescent‐specific mental health services are very limited in Thailand [46, 47]. Another possible explanation is that since we excluded participants who had already received treatment/intervention for MHDs from this study, our prevalence, particularly for AYHIV, might be biased toward a low prevalence. In addition, for the small group of AYHIV with behaviourally acquired infection, we had limited power to demonstrate the differences in prevalence of significant depressive and anxiety symptoms, or suicidality in comparison to HUAY.

In this study, urban residence increased the risk of elevated mental health screening scores among AYHIV, whereas older age decreased the risk. A previous study in urban Uganda noted similar findings in which younger adolescents were at higher risk of psychological illnesses, evaluated by the self‐rating questionnaire 25 (SRQ‐25) [30]. Although it was unclear, we hypothesized that residing in urban environments increased risk of elevated mental health screening scores in AYHIV because of physical environments (e.g. high pollution, traffic) and social environments (e.g. low social support, social segregation), resulting in poorer mental health outcomes compared with those living in rural areas [48]. This finding conflicts with the US study which demonstrated that men living with HIV residing in rural areas had significantly higher risk of depression than urban residing men [49].

All AYHIV with psychiatrist‐confirmed MHDs received mental health services, including mental health counselling, psychotropic medications and psychotherapy. The main prescribed psychiatric medication for MDD, anxiety disorders, PDD and PTSD were SSRIs (e.g. sertraline, fluoxetine), and benzodiazepines (e.g. lorazepam). All are listed in the Thailand National List of Essential Medicines [50], and can be prescribed by general practitioners. These psychotropic medications conformed to guidelines for MHD management in youth living with HIV recommended by the Southern African HIV Clinicians Society (SAHCS), which advises youth with moderate to severe depression and anxiety disorders be initiated on SSRIs in addition to psychosocial management [51]. Additionally, for youth with suicidality, SAHCS recommends the assessment of suicide risk severity, development of a safety plan and hospitalization for high‐risk patients [51]. This practice was carried out in this study.

With appropriate mental health services, 42% of our AYHIV with psychiatrist‐confirmed MHDs exhibited an absence of significant mental health symptoms from the reassessments, and 26% showed an improved score at one‐year follow‐up. This finding seems to be lower than which observed in previous studies that episodes of depression and anxiety in youth usually remit within a year after appropriate treatment [52, 53]. Our suboptimal treatment responses might be because some of our AYHIV had complex mental illnesses which might take longer treatment duration to observe an improvement. This underscores the need for a collaborative integrative model of HIV and mental health services for AYHIV, particularly in resource‐limited settings where a mismatch between the burden of MHDs and the availability of mental health resources has increased tremendously.

The strengths of this study include the availability of psychiatrist‐confirmed diagnoses in participants with elevated mental health screening scores, and a high retention of study participants. However, as MHDs are dynamic conditions with varying degrees of illnesses, a single screening assessment would not sufficiently detect the full spectrum of mental illnesses in patients. Thus, we were not able to exclude MHDs among participants who demonstrated an absence of significant mental health symptoms at enrolment since we did not refer them to a psychiatrist for confirmation. Additionally, we had limited ability to comment on the change of their mental health status over time as we did not repeat an evaluation at one‐year visit. Since diagnostic assessments were only performed on participants with elevated mental health screening scores, the sensitivity of screening measures cannot be evaluated. Furthermore, as we classified participant’s sexual orientation as heterosexual vs. LGBTQ, it might not be appropriate to make a comparison between these two groups as a person can identify as transgender and heterosexual. Recall and reporting bias were inevitable as several variables were self‐reported. In Thai culture, there is a reluctance to express true feelings to health professionals, which may have limited our ability to measure the true disease burden, especially for conditions that manifest as internalization issues more than external behavioural problems. This emphasizes the need for HIV providers to initiate conversations on mental health and actively screen for MHDs even when symptoms are not obviously apparent.

## 5 CONCLUSIONS

With the significant burden of MHDs among AYHIV, mental health screening with simple tools, together with provision of mental health care with well‐established psychiatric consultation and referral pathways at HIV healthcare facilities are necessary. Importantly, a collaborative integrative model of mental health and HIV services would be key to successful comprehensive care for AYHIV.

## COMPETING INTEREST

All authors declare no conflict of interest related to this study.

## AUTHORS’ CONTRIBUTIONS

TS developed the conception of research, designed the study and developed the protocol. TS, LA, WS, TC, RN, PJ, CS and TP conducted the clinical study. AN and PT performed psychiatric evaluations, diagnosed mental health disorders and provided appropriate mental health services. TS conducted the statistical analyses and contributed to the interpretation of study results. TS wrote the first draft of manuscript. TP and WS provided revisions to the manuscript. All authors reviewed and approved the final version of the manuscript.

## ABBREVIAITIONS

aOR, adjusted odds ratio; AUDIT, the Alcohol Use Disorders Identification Test; AYHIV, adolescents and young adults living with HIV; BSI, the Brief Symptom Inventory (BSI); cART, combination antiretroviral treatment; CDI:S, the Children’s Depression Inventory Short Form; CDRS‐R, the Children’s Depression Rating Scale, Revised; CMU, Chiang Mai University; CU, Chulalongkorn University; DSM‐IV, the Diagnostic and Statistical Manual of Mental Disorders, 4th edition; GAD‐7, Generalized Anxiety Disorder 7‐item Scale; HUAY, HIV‐uninfected adolescents and young adults; IQR, interquartile range; LGBTQ, lesbian, gay, bisexual, transgender or queer; MDD, major depressive disorder; MHDs, Mental health disorders; MINI Kid, the Mini‐International Neuropsychiatric Interview for Children and Adolescents; OR, odds ratio; PDD, persistent depressive disorder; PHQ‐9, Patient Health Questionnaire‐9; PLHIV, people living with HIV; PTSD, post‐traumatic stress disorder; RCMAS, the Revised Children’s Manifest Anxiety Scale; SAHCS, the Southern African HIV Clinicians Society; SRQ‐25, the self‐rating questionnaire 25; SSRI, selective serotonin reuptake inhibitors; US, the United States; 95% CI, 95% confidence interval.

## Supporting information


**Figure S1.** The details of provided mental health services, clinical course during study follow‐up, and mental health outcomes after psychiatric referral of study participants, stratified by type of mental health disorder.

**Table S1.** Study participants with mental health disorders and the mental health outcomes at one year follow‐up after psychiatric referral
