# Supplementary material for: Integration of mental health services into HIV healthcare facilities among Thai adolescents and young adults living with HIV
Source: J Int AIDS Soc. 2021 Feb 10;24(2):e25668. doi: 10.1002/jia2.25668 (PMC7876472; doi:10.1002/jia2.25668)
Supplement: Supplementary file 1 — Figure S1. The details of provided mental health services, clinical course during study follow‐up, and mental health outcomes after psychiatric referral of study participants, stratified by type of mental health disorder. [file JIA2-24-e25668-s001.docx]

**Supplemental Figure 1. The details of provided mental health services, clinical course during study follow-up, and mental health outcomes after psychiatric referral of study participants, stratified by type of mental health disorder**


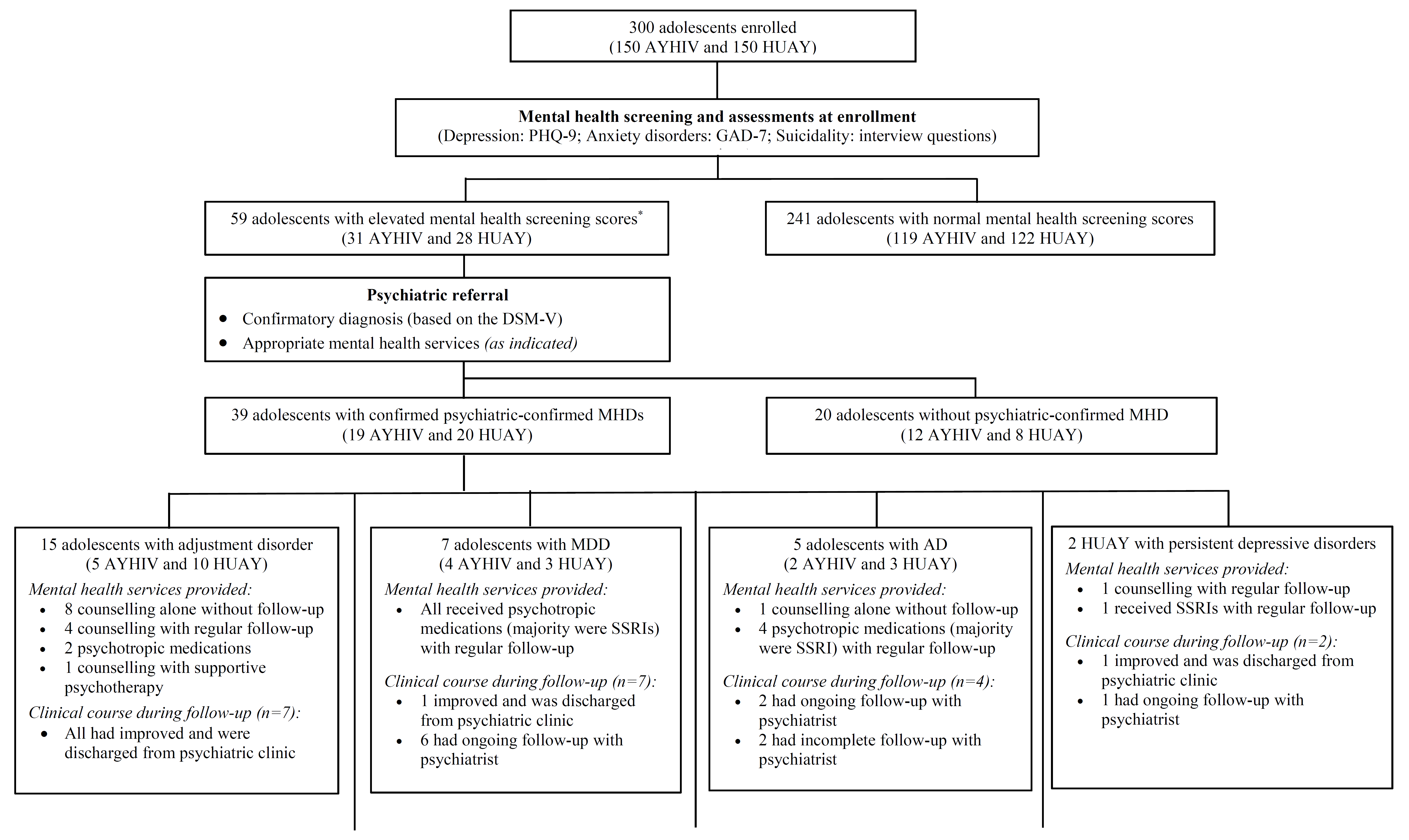


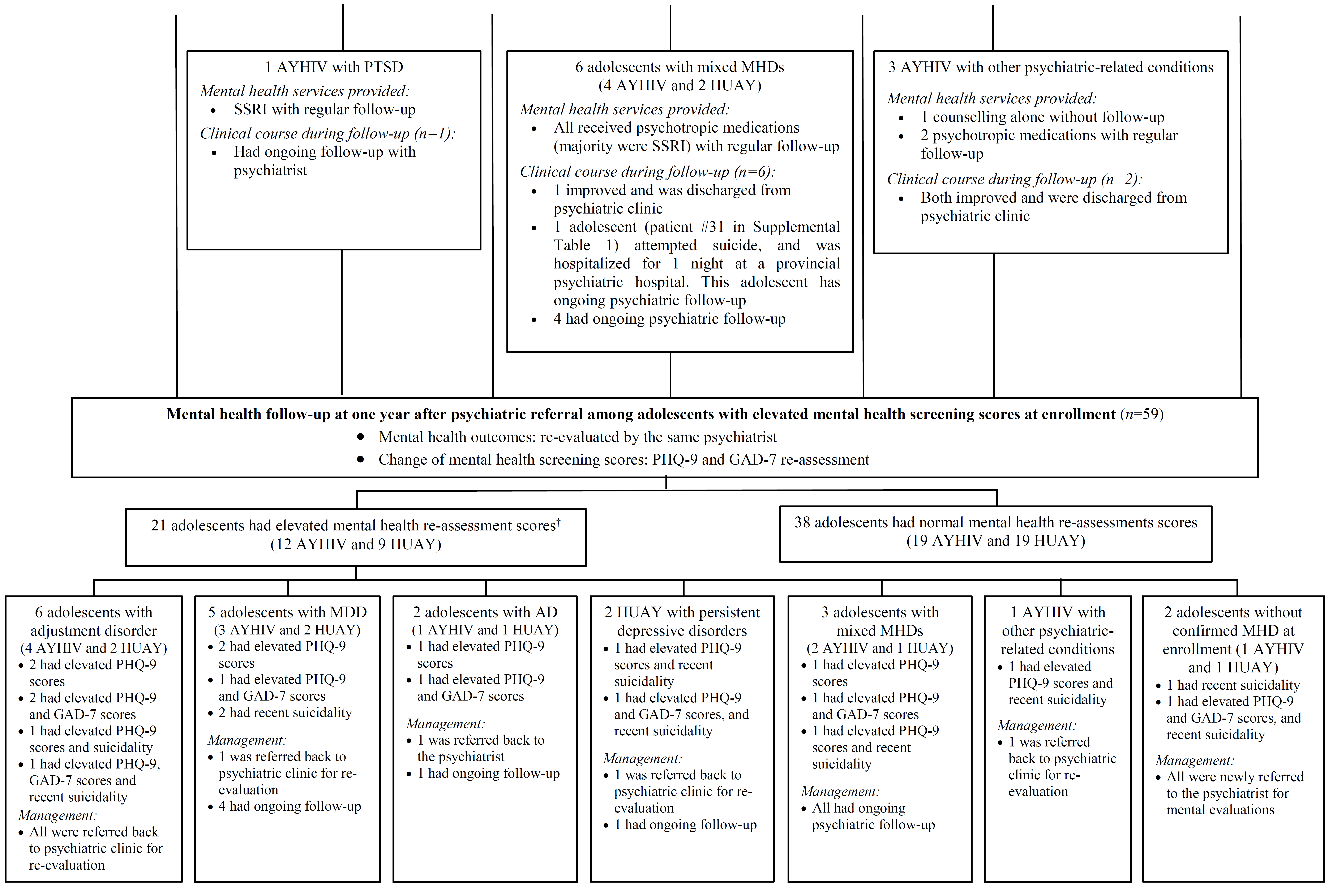


Abbreviations: AYHIV, adolescents and young adults living with HIV; DSM-V, the Diagnostic and Statistical Manual of Mental Disorders criteria, 5^th^ edition; GAD-7, the Generalized Anxiety Disorder 7-item scales; HUAY, HIV-uninfected adolescents and young adults; MDD, major depressive disorder; MHDs, mental health disorders; PHQ-9, the Patient Health Questionnaire 9-item; PTSD, post-traumatic stress disorders.

^*^Elevated mental health screening scores was defined as having either significant depressive symptoms (PHQ-9 ≥9), significant anxiety symptoms (GAD-7 ≥10), or suicidal behaviors (ever had suicidal ideation and/or attempted suicide during lifetime, or had recent suicidal thought within the past 2 weeks [responded positively to PHQ-9, question #9]).

^†^Elevated mental health re-assessment scores was defined as having either significant depressive symptoms (PHQ-9 ≥9, significant anxiety symptoms (GAD-7 ≥10), or recent suicidal thought within the past 2 weeks (responded positively to PHQ-9, question #9).
