# Supplementary material for: Integration of mental health services into HIV healthcare facilities among Thai adolescents and young adults living with HIV
Source: J Int AIDS Soc. 2021 Feb 10;24(2):e25668. doi: 10.1002/jia2.25668 (PMC7876472; doi:10.1002/jia2.25668)
Supplement: Supplementary file 2 — Table S1. Study participants with mental health disorders and the mental health outcomes at one year follow‐up after psychiatric referral [file JIA2-24-e25668-s002.docx]

**Supplemental Table 1. Study participants with mental health disorders and the mental health outcomes at one year follow-up after psychiatric referral**

| **No.** | **Age**  (years) | **Sex** | **HIV status** | **Current cART regimen** | **Mental health screening at enrollment** | | | | **Psychiatrist-confirmed psychiatric disorder**  **(DSM-V)** | **Psychiatric treatment and intervention** | **Mental health outcomes at one year follow-up** | | | |  | **Mental health screening at one year follow-up** | | |
| --- | --- | --- | --- | --- | --- | --- | --- | --- | --- | --- | --- | --- | --- | --- | --- | --- | --- | --- |
|  |  |  |  |  | **PHQ-9 score** | **PHQ-9**  **item #9**  (Y/N) | **GAD-7 score** | **Lifetime suicidality** (Y/N) |  |  | **Treatment status** | **Treatment outcome** | **Treatment duration** (days) | **Number of visit(s) with psychiatrist** |  | **PHQ-9 score** | **PHQ-9 item #9**  (Y/N) | **GAD-7 score** |
| ***Adjustment disorder (n = 15)*** | | | | | | | | | | | | | | | | | | |
| 1 | 16.6 | M | AYHIV | PI | 14 | Y | 6 | NR | AjD | Counselling | - | - | - | 1 |  | 9 | N | 5 |
| 2 | 21.3 | M | AYHIV | NNRTI | 10 | N | 2 | N | AjD | Counselling | Stop | Improved | 168 | 2 |  | 7 | N | 3 |
| 3 | 17.0 | F | AYHIV | PI | 16 | Y | 10 | Y | AjD | Counselling | Stop | Improved | 59 | 2 |  | 13 | N | 14 |
| 4 | 19.3 | F | AYHIV | PI | 8 | N | 4 | Y | AjD | Zolpidem tartrate | Stop | Improved | 63 | 2 |  | 9 | N | 4 |
| 5 | 22.7 | F | AYHIV | PI | 10 | N | 7 | N | AjD | Mirtazapine | Stop | Improved | 350 | 6 |  | 12 | Y | 6 |
| 6 | 17.9 | M | HUAY | - | 10 | Y | 12 | Y | AjD | Counselling | - | - | - | 1 |  | 0 | N | 1 |
| 7 | 22.3 | M | HUAY | - | 6 | N | 5 | Y | AjD | Counselling | Stop | Improved | 168 | 2 |  | 6 | N | 4 |
| 8 | 25.0 | M | HUAY | - | 12 | Y | 11 | Y | AjD | Counselling | - | - | - | 1 |  | 2 | N | 0 |
| 9 | 15.3 | F | HUAY | - | 11 | N | 7 | Y | AjD | Counselling | - | - | - | 1 |  | 4 | N | 6 |
| 10 | 15.8 | F | HUAY | - | 17 | Y | 16 | Y | AjD | Counselling | - | - | - | 1 |  | 20 | Y | 18 |
| 11 | 16.8 | F | HUAY | - | 5 | Y | 6 | Y | AjD | Counselling | - | - | - | 1 |  | 3 | N | 3 |
| 12 | 17.6 | F | HUAY | - | 17 | N | 15 | N | AjD | Counselling | - | - | - | 1 |  | 7 | N | 2 |
| 13 | 17.6 | F | HUAY | - | 18 | N | 10 | N | AjD | Counselling, Supportive psychotherapy | Stop | Improved | 38 | 3 |  | 12 | N | 14 |
| 14 | 19.6 | F | HUAY | - | 3 | N | 3 | Y | AjD | Counselling | Stop | Improved | 67 | 2 |  | 6 | N | 0 |
| 15 | 21.6 | F | HUAY | - | 14 | N | 8 | N | AjD | Counselling | - | - | - | 1 |  | 8 | N | 3 |
| ***Major depressive disorder (n = 7)*** | | | | | | | | | | | | | | | | | | |
| 16 | 16.6 | M | AYHIV | PI | 7 | Y | 10 | Y | MDD | Sertraline, Fluoxetine, Risperidone | Ongoing | Ongoing | Ongoing | 9 |  | 10 | N | 11 |
| 17 | 15.4 | F | AYHIV | NNRTI | 21 | Y | 15 | Y | MDD | Escitalopram | Stop | Improved | 273 | 5 |  | 16 | N | 8 |
| 18 | 16.4 | F | AYHIV | PI | 6 | N | 4 | Y | MDD | Amitriptyline | Ongoing | Ongoing | Ongoing | 5 |  | 5 | N | 6 |
| 19 | 18.5 | F | AYHIV | INSTI | 7 | Y | 2 | N | MDD | Trazodone | Ongoing | Ongoing | Ongoing | 2 |  | 13 | N | 9 |
| 20 | 15.3 | F | HUAY | - | 10 | N | 4 | N | MDD | Sertraline | Ongoing | Ongoing | Ongoing | 3 |  | 6 | N | 4 |
| 21 | 15.4 | F | HUAY | - | 2 | N | 1 | Y | MDD | Escitalopram | Ongoing | Ongoing | Ongoing | 7 |  | 7 | Y | 1 |
| 22 | 16.6 | F | HUAY | - | 11 | N | 8 | Y | MDD | Sertraline, Lorazepam | Ongoing | Ongoing | Ongoing | 10 |  | 8 | Y | 6 |
| ***Anxiety disorders (n = 5)*** | | | | | | | | | | | | | | | | | | |
| 23 | 22.6 | M | AYHIV | PI | 8 | N | 12 | Y | AD | Sertraline, Propranolol | Ongoing | Ongoing | Ongoing | 7 |  | 14 | N | 12 |
| 24 | 20.9 | F | AYHIV | PI | 5 | N | 4 | Y | AD | Escitalopram | Ongoing | Ongoing | Ongoing | 6 |  | 4 | N | 3 |
| 25 | 17.1 | M | HUAY | - | 9 | N | 5 | N | AD | Counselling | - | - | - | 1 |  | 8 | N | 6 |
| 26 | 20.6 | M | HUAY | - | 12 | N | 10 | N | AD | Sertraline | Stop | Incomplete follow-up | 289 | 10 |  | 15 | N | 7 |
| 27 | 23.2 | M | HUAY | - | 5 | Y | 4 | Y | AD | Sertraline, Lorazepam | Stop | Incomplete follow-up | 225 | 2 |  | 2 | N | 1 |
| ***Persistent depressive disorder (n = 2)*** | | | | | | | | | | | | | | | | | | |
| 28 | 15.4 | F | HUAY | - | 9 | N | 2 | N | PDD | Counselling | Stop | Improved | 267 | 7 |  | 17 | Y | 13 |
| 29 | 20.1 | F | HUAY | - | 11 | Y | 7 | Y | PDD | Sertraline, Fluoxetine | Ongoing | Ongoing | Ongoing | 8 |  | 10 | Y | 2 |
| ***Post-traumatic stress disorder (n = 1)*** | | | | | | | | | | | | | | | | | | |
| 30 | 15.1 | F | AYHIV | PI | 6 | Y | 4 | N | PTSD | Sertraline | Ongoing | Ongoing | Ongoing | 5 |  | 4 | N | 1 |
| ***Mixed mental health disorders (n = 6)*** | | | | | | | | | | | | | | | | | | |
| 31^*^ | 16.2 | M | AYHIV | NNRTI | 4 | N | 1 | Y | MDD and PTSD | Sertraline, Lorazepam, Escitalopram | Ongoing | Ongoing | Ongoing | 13 |  | 9 | N | 8 |
| 32 | 20.4 | M | AYHIV | INSTI | 16 | Y | 18 | Y | MDD, AD and PTSD | Sertraline, Lorazepam | Ongoing | Ongoing | Ongoing | 9 |  | 9 | N | 10 |
| 33 | 24.4 | M | AYHIV | INSTI + PI | 11 | N | 6 | N | MDD and AD | Sertraline, Lorazepam | Stop | Improved | 154 | 4 |  | 7 | N | 5 |
| 34 | 18.9 | F | AYHIV | NNRTI | 11 | N | 5 | N | MDD and PTSD | Sertraline, Mirtazapine | Ongoing | Ongoing | Ongoing | 11 |  | 2 | N | 0 |
| 35 | 20.7 | M | HUAY | - | 6 | Y | 3 | Y | MDD and AD | Sertraline, Risperidone | Ongoing | Ongoing | Ongoing | 8 |  | 0 | N | 0 |
| 36 | 23.8 | F | HUAY | - | 16 | Y | 11 | Y | MDD and AD | Sertraline, Gabapentin | Ongoing | Ongoing | Ongoing | 6 |  | 13 | Y | 5 |
| ***Other psychiatric-related conditions (n = 3)*** | | | | | | | | | | | | | | | | | | |
| 37 | 20.6 | M | AYHIV | NNRTI | 15 | Y | 12 | Y | Intellectual disability | Lorazepam | Stop | Improved | 146 | 4 |  | 6 | N | 4 |
| 38 | 21.1 | M | AYHIV | NNRTI | 9 | N | 3 | N | Insomnia | Lorazepam | Stop | Improved | 81 | 4 |  | 11 | Y | 7 |
| 39 | 15.4 | F | AYHIV | NNRTI | 5 | Y | 4 | Y | Parent child relational problem | Counselling | - | - | - | 1 |  | 3 | N | 3 |
| ***Normal mental health evaluation (n = 20)*** | | | | | | | | | | | | | | | | | | |
| 40 | 15.2 | M | AYHIV | NNRTI | 14 | N | 5 | N | Normal | Counselling | - | - | - | 1 |  | 5 | N | 1 |
| 41 | 16.7 | M | AYHIV | NNRTI | 20 | Y | 11 | Y | Normal | Counselling | - | - | - | 1 |  | 15 | Y | 10 |
| 42 | 17.5 | M | AYHIV | NNRTI | 4 | N | 1 | Y | Normal | Counselling | - | - | - | 1 |  | 3 | N | 2 |
| 43 | 17.9 | M | AYHIV | PI | 5 | N | 10 | N | Normal | Counselling | - | - | - | 1 |  | 2 | N | 2 |
| 44 | 18.2 | M | AYHIV | INSTI | 9 | Y | 13 | Y | Normal | Counselling | - | - | - | 1 |  | 5 | N | 4 |
| 45 | 19.7 | M | AYHIV | NNRTI | 14 | N | 10 | N | Normal | Counselling | - | - | - | 1 |  | 0 | N | 0 |
| 46 | 20.9 | M | AYHIV | NNRTI | 9 | N | 0 | N | Normal | Counselling | - | - | - | 1 |  | 4 | N | 0 |
| 47 | 15.5 | F | AYHIV | NNRTI | 10 | Y | 13 | N | Normal | Counselling | - | - | - | 1 |  | 7 | N | 4 |
| 48 | 16.8 | F | AYHIV | NNRTI | 3 | N | 3 | Y | Normal | Counselling | - | - | - | 1 |  | 3 | N | 3 |
| 49 | 17.1 | F | AYHIV | PI | 6 | N | 4 | Y | Normal | Counselling | - | - | - | 1 |  | 3 | N | 4 |
| 50 | 17.3 | F | AYHIV | INSTI | 10 | N | 3 | N | Normal | Counselling | - | - | - | 1 |  | 2 | N | 0 |
| 51 | 17.9 | F | AYHIV | NNRTI | 2 | Y | 1 | Y | Normal | Counselling | - | - | - | 1 |  | 0 | N | 1 |
| 52 | 16.0 | M | HUAY | - | 11 | Y | 8 | N | Normal | Counselling | - | - | - | 1 |  | 7 | N | 4 |
| 53 | 16.5 | M | HUAY | - | 6 | Y | 6 | N | Normal | Counselling | - | - | - | 1 |  | 2 | N | 4 |
| 54 | 21.4 | M | HUAY | - | 5 | Y | 8 | N | Normal | Counselling | - | - | - | 1 |  | 3 | N | 3 |
| 55 | 23.8 | M | HUAY | - | 1 | Y | 6 | Y | Normal | Counselling | - | - | - | 1 |  | 1 | N | 2 |
| 56 | 15.6 | F | HUAY | - | 7 | Y | 2 | N | Normal | Counselling | - | - | - | 1 |  | 7 | Y | 6 |
| 57 | 15.6 | F | HUAY | - | 11 | N | 11 | N | Normal | Counselling | - | - | - | 1 |  | 3 | N | 2 |
| 58 | 21.8 | F | HUAY | - | 14 | N | 6 | N | Normal | Counselling | - | - | - | 1 |  | 5 | N | 1 |
| 59 | 22.6 | F | HUAY | - | 7 | N | 1 | Y | Normal | Counselling | - | - | - | 1 |  | 6 | N | 3 |

Abbreviations: AD, anxiety disorder; AjD, adjustment disorder; AYHIV, adolescent and young adult living with HIV; DSM-V, The Diagnostic and Statistical Manual of Mental Disorders, 5th edition; F, female; GAD-7, the Generalized Anxiety Disorder 7-item scales; HUAY, HIV-uninfected adolescent and young adult; INSTI, integrase strand transfer inhibitor; M, male; MDD, major depressive disorder; N, no; NNRTI, non-nucleoside reverse transcriptase inhibitor; NR, no response; PDD, persistent depressive disorder; PHQ-9, the Patient Health Questionnaire 9-item; PI, protease inhibitor; PTSD, post-traumatic stress disorder; Y, yes.

^*^This adolescent had a suicidal attempt during their treatment course and was hospitalized for 1 night at a provincial psychiatric hospital.
